# The Consistency of *CDC73* Mutation and Parafibromin Staining Loss in Parathyroid Neoplasm: A Systematic Review

**DOI:** 10.1155/ije/1905585

**Published:** 2025-08-20

**Authors:** Jinheng Xiao, Sen Yang, Qingyuan Zheng, Tianqi Chen, Ya Hu

**Affiliations:** ^1^Department of General Surgery, Peking Union Medical College Hospital, Chinese Academy of Medical Sciences & Peking Union Medical College, Beijing, China; ^2^Medical Research Center, Peking Union Medical College Hospital, Chinese Academy of Medical Sciences & Peking Union Medical College, Beijing, China

**Keywords:** cancer, *CDC73*, parafibromin, parathyroid neoplasm

## Abstract

**Background:** Primary hyperparathyroidism (pHPT) caused by parathyroid neoplasm is a common endocrine disorder. Nuclear staining loss of parafibromin, encoded by the *CDC73* gene, has been shown to be closely related to parathyroid malignancy and poor prognosis. Although previous studies have found that parafibromin staining loss is not always consistent with *CDC73* mutation, the reasons are still unknown.

**Methods:** Published studies from the PubMed database were searched using the terms “parafibromin,” “*CDC73*,” “*HRPT2*,” and “parathyroid” to identify eligible studies. Among the included studies, *CDC73* mutation and parafibromin immunohistochemical (IHC) results for patients with parathyroid neoplasms were reviewed, and possible reasons for the inconsistency between the parafibromin staining loss and *CDC73* mutation were explored. This systematic review was conducted following the Preferred Reporting Items for Systematic Reviews and Meta-Analyses (PRISMA) 2020 analysis protocol.

**Results:** A total of 299 patients from 32 studies were included in the present review. Inconsistency and consistency between parafibromin staining and *CDC73*status was observed in 19.40% and 80.60% of patients. Patients in the inconsistency group showed higher level of serum calcium (*p* = 0.026). Significant difference in the inconsistency rate was found between PC (25.15%) and non-PC group (12.50%) (*p* < 0.001), and NGS (8.51%) and non-NGS group (21.43%) (*p* = 0.006) in multivariate analysis.

**Conclusion:** The main reasons for the inconsistency were attributed to the pathological type and sequencing method. More inconsistent results were detected in the PC group and the non-NGS group.

## 1. Introduction

Primary hyperparathyroidism (pHPT) is a common endocrine disorder caused by excessive parathyroid hormone (PTH) secreted from hyperplastic parathyroid tissues. The main clinical presentations of pHPT include hypercalcemia, urinary calculus, renal insufficiency, osteoporosis, bone fracture, and even hypercalcemia crisis. Most pHPTs are caused by sporadic neoplasms, whereas up to 10% of patients have a familial history of pHPT or harbor germline mutations [1]. Most sporadic neoplasms are parathyroid adenoma (PA), with parathyroid carcinoma (PC) accounting for approximately 1% of pHPT cases [2]. According to WHO classification, diagnosis of PC should be based on the following evidence: local invasion into surrounding tissue, angioinvasion, lymphatic invasion, perineural (or intraneural) invasion, or histologically confirmed metastasis [3]. Atypical parathyroid tumors (APTs) are parathyroid neoplasms with atypical histological features and uncertain malignant potential [3].

*CDC73*, mapping at 1q31.2, contains 17 exons that encode the 531 amino acids of the protein termed parafibromin. Inactivating mutations in *CDC73* were first discovered in hyperparathyroidism-jaw tumor syndrome (HPT-JT), an autosomal dominant genetic disease featuring parathyroid tumors with fibro-osseous jaw tumors as well as uterine and renal lesions [4]. Then, *CDC73* mutations are found in a variety of tumors, including gastric cancer, colorectal cancer, and ovarian cancer [5]. *CDC73* mutations have been identified in up to 75% of PC patients [6]. Dysregulated expression of *CDC73* correlates with aggressive behavior of malignancies and unfavorable prognosis of PC [5]. Approximately 80% of *CDC73* mutations are located in exons 1, 2, and 7 [5]. *CDC73* mutations may result in defective function or truncation of the parafibromin peptide, leading to loss of parafibromin expression in the nucleus of tumor cells. Parafibromin contains two recognized protein domains, the N-terminal domain (NTD) and C-terminal domain (CTD) [7]. As a part of the polymerase-associated factor 1 (PAF1) complex, parafibromin regulates gene translation by interacting with RNA polymerase II. Furthermore, parafibromin plays an important role in cell motility, apoptosis, and proliferation, among others [7].

Parafibromin is not only regarded as diagnostic biomarker for PC but also reported to be closely related to the recurrence, metastasis, and mortality of PC [8–10]. However, the synergistic application of molecular screening and IHC is essential to achieve optimal diagnostic efficacy [11, 12]. Moreover, the relationship between *CDC73* mutation and prognosis of PC has not been determined definitely. Thus, the *CDC73* status plays a vital role in the diagnosis and management of parathyroid tumors. In some previous studies, the consistency of *CDC73* mutation and parafibromin staining was up to 100% [13–15]. Based on next-generation sequencing (NGS) and parafibromin staining of 23 PCs, our previous studies found high consistency between *CDC73* and parafibromin and suggested that *CDC73* status may also play a prognostic role [16]. However, Cetani [16] and Zhu et al. [9] reported discrepant results of *CDC73* sequencing and parafibromin IHC staining in 2014 and 2020, respectively. Hence, the purpose of this article was to review reported parathyroid neoplasm cases with both results of *CDC73* sequencing and parafibromin staining from previous publications. Possible reasons for the inconsistency between *CDC73*statusand parafibromin staining are explored for further clinical application.

## 2. Materials and Methods

### 2.1. Search Strategies

The PubMed database was searched to identify eligible studies. The following keywords, “parafibromin” OR “*CDC73*” OR “*HRPT2*” AND “parathyroid”, were searched in keywords and abstracts. The reference lists of eligible articles were also screened for additional studies.

### 2.2. Study Selections

**Figure 1:**
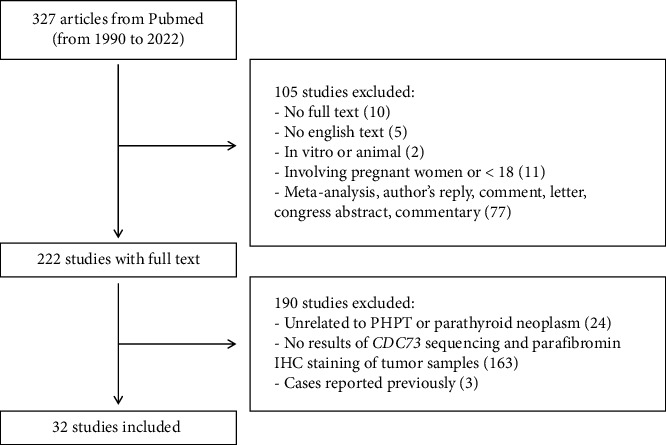
The flowchart of the search and selection procedure for the included studies.

Two researchers screened the literature independently and reached a consensus. A study was included if it met all of the following criteria: (1) patients in the study were diagnosed with pHPT clinically and diagnosed with PA, PC, parathyroid hyperplasia, or APT histologically according to the WHO 2017 criteria [17]or WHO 2004 criteria [18]; (2) results of *CDC73* sequencing and parafibromin IHC staining of tumor samples could be extracted. Studies were excluded if they were as follows: (1) parafibromin IHC staining of tumor tissue was absent or only WBC (white blood cell) DNA was sequenced without identified mutation; (2) in vitro or animal studies; (3) a meta-analysis, review, author's reply, letter, comment or conference abstract; (4) the age of patients < 18 or pregnant (Figure 1). If patients were repeatedly reported in different articles, the most detailed or the latest version was chosen.

### 2.3. Sequencing Methods

Sanger sequencing, also known as the chain-termination method, is a method that identifies the order of nucleotide bases in DNA based on chain termination by modified nucleotides called dideoxynucleotide triphosphates (ddNTPs). Multiplex ligation-dependent probe amplification (MLPA) is a technique that detects copy number variations and point mutations in DNA based on polymerase chain reaction (PCR). NGS, also known as high-throughput sequencing, is a robust platform that can sequence millions of DNA fragments simultaneously.

### 2.4. Data Extraction

The following information was extracted from the included studies independently by two reviewers: patient age, sex, serum calcium and PTH level, gene sequencing methods, the parafibromin antibody used for IHC, scoring criteria of parafibromin IHC, results of *CDC73* sequencing, results of parafibromin staining, the first author, and year of publication.

### 2.5. Statistical Analysis

Continuous variables are presented as the mean value ± standard deviation (SD). Categorical variables are described as case numbers or corresponding percentages. Differences among patient groups were tested by *χ*^2^ tests and *t*-tests, as appropriate for categorical variables and continuous variables, respectively. A value of *p* < 0.05 was regarded as statistically significant. All statistical analyses were performed with SPSS Statistics for Windows Version 16 (SPSS Inc., Chicago, IL, USA).

## 3. Results

### 3.1. Eligible Articles and Patients

A total of 299 patients from 32 publications were included in this review [10–16, 19–43] (Figure 1) The basic information of included studies and characteristics of the included patients are shown in Tables 1 and 2, respectively. Among these patients, the male-to-female ratio was 0.895, and the mean age was 47.91 ± 16.95 years (range 18–86 years). The mean serum total calcium (normal range: 2.15–2.65 mmol/L) and intact PTH (normal range: 15–65 pg/mL) at first diagnosis were 3.28 ± 0.55 mmol/L (range 1.88–5.05) and 620.54 ± 703.21 pg/mL (range 55.0–3875.0), respectively. Of the 299 cases, PC, PA, and APT accounted for 54.52%, 39.13%, and 6.35%, respectively.

### 3.2. *CDC73* Mutation and Parafibromin Staining Loss

Parafibromin staining loss and *CDC73* mutation were identified in 56.19% and 53.51% of patients, respectively. A total of 160 patients harbored *CDC73* mutation, with germline mutations in 74 patients, somatic mutations in 57 patients, germline and somatic mutations in 26 patients, and unknown mutation types in 3 patients. In addition, 6 patients harbored two somatic mutations. A total of 168 cases were determined to have negative parafibromin staining, and 131 cases were determined to have positive staining. Inconsistency of both results was identified in 58 (19.40%) cases in 20 studies. Patients who harbored *CDC73* mutation but showed positive parafibromin staining were identified as *CDC73* (+)/parafibromin (+), and patients who had no *CDC73* mutation but showed negative parafibromin staining were identified as *CDC73* (−)/parafibromin (−). A total of 25 patients were *CDC73* (+)/parafibromin (+), and 33 patients were *CDC73* (−)/parafibromin (−). A significant difference (*p*=0.026) in serum calcium levels was showed between the consistency group (3.24 ± 0.54 mmol/L) and the inconsistency group (3.48 ± 0.58 mmol/L).

### 3.3. Possible Reasons for Inconsistent Results Between *CDC73* Status and Parafibromin IHC Staining

#### 3.3.1. Pathologic Diagnosis

In the PC group and non-PC group, the proportions of patients with inconsistent results were 25.15% and 12.50%, respectively (*p*=0.006) (Table 3). Among 58 patients with inconsistent results, PC accounted for 70.69% (41/58), which was higher than non-PC cases (17/58, 29.31%). Among PC patients with inconsistent results, *CDC73* (+)/parafibromin (+) and *CDC73* (−)/parafibromin (−) accounted for 39.02% (16/41) and 60.98% (25/41), respectively.

#### 3.3.2. Sequencing Methods

Among the 24 studies that employed non-NGS, MLPA was utilized in 2 studies, and NGS was applied in 6 other studies. Of the 47 patients for whom NGS was performed, 4 (8.51%) patients had inconsistent results, while 54 (21.43%) inconsistent results were found in 252 patients for whom non-NGS was performed. Compared with NGS, more inconsistent results were found in patients with non-NGS (*p* = 0.040) (Table 3).

#### 3.3.3. Parafibromin Antibody and IHC Scoring

Antiparafibromin antibody sc-33638 targeting aa 87–100 (Santa Cruz USA) was used in 21 studies, and other types of antibodies were used in 11 studies. Different criteria were applied for negative parafibromin staining in the studies. In 25 studies, total nuclear IHC staining loss was defined as parafibromin staining loss in IHC scoring, while partial staining loss was used as the negative standard in the other 6 studies and no definite criteria was showed in one study. Antibody type (*p* = 0.288) or scoring criteria (*p* = 0.160) was not found to be related to the consistency rate in univariate analysis.

#### 3.3.4. Somatic Mutation

Among 105 patients with both germline and somatic mutations information, 64 patients harbored somatic mutations, and 29 patients showed two or more sites of mutations. However, the effect of somatic mutation (*p* = 0.460) or multisite mutation (*p* = 0.439) on consistency has not been found.

#### 3.3.5. Family History

A total of 54 patients had a family history, with 85.45% (47/55) being diagnosed with PA. In all patients, there was no correlation between family history and inconsistency (*p*=0.562).

### 3.4. Multivariate Analysis of the Reason for the Inconsistency

With logistic regression, the observed inconsistent result was found to be closely related to the pathologic diagnosis (*p* < 0.001) and sequencing method (*p* = 0.006). Other factors such as the scoring criteria (*p* = 0.946) or the IHC antibody type (*p* = 0.888) were not related to the inconsistent results in multivariate analysis.

## 4. Discussion

In 2004, the Tan team took the lead in developing a parafibromin monoclonal antibody for immunohistochemistry and found that loss of parafibromin immunoreactivity was consistent with somatic *HRPT2* gene mutation in sporadic PC [44]. However, inconsistencies in results of *CDC73* mutation and parafibromin staining have been found in many subsequent studies, and the significance of *CDC73* in predicting prognosis has been questioned [9, 10]. Our present study sought to identify possible reasons for this inconsistency.

In our present study, the sequencing method was found to be an important factor affecting the consistency of *CDC73* mutations and parafibromin staining results. NGS can increase the detection sensitivity of *CDC73* mutations, resulting in a significant decrease in the number of patients with *CDC73* (−)/parafibromin (−). Previous studies have confirmed that the sensitivity of NGS is higher than that of Sanger sequencing [45, 46]. In 2020, the European Society for Medical Oncology (ESMO) recommended the use of NGS for a variety of malignancies in clinical practice [47]. Therefore, NGS should also be prioritized for future clinical research in parathyroid neoplasms.

However, NGS could not resolve all problems such as cases with *CDC73* (−)/parafibromin (−). Of all patients for whom NGS was performed, 3 patients still showed *CDC73* (−)/parafibromin (−). The reason may be that some large fragment deletions cannot be detected by NGS. Detection of large fragment deletions is needed in tumors with aggressive behavior and may further improve the rate of consistency [48]. Other possible reasons may include epigenetic abnormalities beyond genetic mutations. For example, gene methylation or microRNA may affect expression of *CDC73*. PCs are characterized by global hypermethylation, and methylation of the *CDC73* gene promoter has been identified in 2/11 PC patients [49]. Overexpression of Wilms tumor suppressor 1 was found to reduce *CDC73* expression by methylation of the *CDC73* promoter in PC [50].

The reason for the positive staining in the patient with the *CDC73* inactivating mutation may be that some gene mutations may not have a significant impact on the structure or function of parafibromin. Some mutations do not affect the antigenic determinant identified and bound by a specified IHC antibody. Li et al. proposed that *CDC73* mutations could be divided into two types, high-impact and low-impact mutations, and that low-impact mutations may not affect or have unknown effects on expression of parafibromin [7]. *CDC73* mutations that were identified in some specific sites may result in expression of truncated parafibromin [51]. Therefore, some *CDC73* mutations might lead to production of defective parafibromin but do not result in negative staining of parafibromin.

Patients with PC are likely to show inconsistent results, and a possible reason is the higher extent of intratumor heterogeneity in PC compared to PA. Intratumor heterogeneity, including clonal heterogeneity and proteome heterogeneity, has been found in PC. Intratumor heterogeneity of parafibromin IHC in PC was verified by a higher proportion of focal loss [52]. Moreover, the detection scope differs between gene sequencing and IHC staining. Only a limited portion of tumor tissue is used for *CDC73* sequencing, whereas more tissue can be observed in IHC. Intratumor heterogeneity interferes with *CDC73* mutation, causing different expressions of parafibromin in subclones, which affects the overall result of parafibromin IHC.

We found that IHC with the sc-33638 antibody and the scoring criterion with total loss as negative staining were widely used. The effect of antibody or scoring criteria on inconsistency could not be identified in univariate and multivariate analyses. However, our previous meta-analysis indicated that the use of an antibody (sc-33638) and strict IHC scoring criteria have lower sensitivity for diagnosis of PC [53], which may increase the rate of *CDC73* (−)/parafibromin (−). The samples stained by the nonsc-33638 antibody were mainly from Juhlin et al. [19] and our team [43]. A comparison of different antibody of parafibromin may be necessary for optimizing clinical practice.

There were several limitations in this study. Although 299 cases were included in this review, the case number and database were still limited, which may underestimate the role of certain factors in multivariate analysis, such as antibody type and IHC scoring criteria. Second, the results from NGS were limited compared with that with Sanger sequencing, which may introduce bias in the analysis of present study. Third, in a few studies, not all exons can be covered due to the limitations of sequencing methods. Hence, further prospective study with a larger sample size is necessary to identify the significance of IHC staining and DNA sequencing in diagnosis of parathyroid neoplasm.

In summary, NGS may reduce the inconsistency of *CDC73* sequencing and parafibromin staining. NGS should be applied for clinical practice and research studies in PC. Compared to other pathology types, PC is more likely to show inconsistent results. Antibodies with higher specificity and sensitivity are needed to decrease the rate of inconsistent results.

## Figures and Tables

**Table 1:** The basic information of included studies.

No.	*n*	Gender		Age (year)	Ca (mmol/L)	PTH (pg/mL)	Family history	Pathology				Sequencing method	Parafibromin IHC		Consistency		References
		M	F					PA	PC	APT	PH		Antibody (sc-33638)	Scoring criteria	Yes	No	
1	41	14	27	64.95	2.90	168.65	0	41	0	0	0	1	0	Negative/weak positive/positive	39	2	2006-Juhlin et al. [19]
2	5	1	1	38.00	—	—	3	3	2	0	0	1	0	0%	4	1	2006-Gill et al. [20]
3	5	3	2	58.67	4.01	—	0	0	5	0	0	1	0	10%	0	5	2007-Juhlin et al. [21]
4	7	3	4	51.14	—	—	0	0	7	0	0	1	0	Global/focal loss	4	3	2007 Haven et al. [22]
5	1	1	0	21.00	4.32	3170.00	0	1	0	0	0	1	0	0%	1	0	2007-Cetani et al. [23]
6	8	3	5	29.25	3.98	1044.50	8	6	1	1	0	1	0	Diffuse loss	8	0	2008-Sarquis et al. [13]
7	14	3	11	37.79	3.11	153.60	14	12	1	1	0	1	1	10%	10	4	2008-Masi et al. [24]
8	2	2	0	43.00	3.43	206.00	0	1	1	0	0	1	0	No staining	2	0	2008-Cetani et al. [25]
9	6	—	—	—	—	—	4	4	2	0	0	1	1	Completely absent	5	1	2009 Howell et al. [26]
10	4	0	4	43.00	3.64	499.75	0	0	4	0	0	1	1	0%	4	0	2010 Corbetta et al. [14]
11	23	11	12	51.78	—	—	0	0	23	0	0	1	0	Global/focal	13	10	2011-Witteveen et al. [27]
12	1	0	1	18.00	3.63	621.00	1	1	0	0	0	1	1	Complete absent	1	0	2011 Cascon et al. [12]
13	2	0	2	28.50	3.13	467.50	0	0	2	0	0	1	1	1%	1	1	2011 Cavaco et al. [28]
14	13	10	3	49.23	3.84	1453.40	0	0	13	0	0	1	1	0%/+/++/+++	3	10	2012-Wang et al. [29]
15	42	14	28	52.95	3.07	386.81	3	17	12	13	0	1	1	Absent	36	6	2012-Guarnieri et al. [11]
16	31	15	16	54.06	3.41	718.56	0	0	31	0	0	1	1	5%	25	6	2013-Cetani et al. [10]
17	4	2	2	22.00	2.89	2019.33	4	3	0	0	1	1	1	0%/+/++/+++	2	2	2014-Kong et al. [30]
18	2	2	0	25.00	1.88	693.00	2	0	2	0	0	1	1	0%	2	0	2014-Korpi-Hyövälti et al. [31]
19	2	2	0	24.50	—	—	0	0	1	1	0	2	1	1%	1	1	2017-Ryhänen et al. [32]
20	1	1	0	35.00	—	—	0	1	0	0	0	1	1	Positive/negative	1	0	2017-Hattangady et al. [33]
21	1	0	1	32.00	2.60	162.00	1	1	0	0	0	2	1	—	0	1	2017-Rubinstein et al. [34]
22	1	0	1	37.00	—	—	1	1	0	0	0	1	0	0%	1	0	2017-van der Tuin et al. [35]
23	1	1	0	25.00	3.95	145.60	0	0	1	0	0	2	1	Global/focal	1	0	2018-Kapur et al. [36]
24	1	0	1	43.00	3.70	2847.00	0	0	1	0	0	1	1	Did not show	1	0	2018-Mahajan and Sacerdote [37]
25	23	15	8	38.00	3.18	277.70	0	13	10	0	0	1	1	0%	23	0	2019-Gill et al. [15]
26	6	3	3	34.33	—	—	5	3	2	1	0	1	1	10%	5	1	2019-Juhlin et al. [38]
27	3	2	1	35.00	3.50	327.00	3	1	2	0	0	1	1	Negative/weak positive/positive	2	1	2019-Grigorie [39]
28	1	1	0	41.00	3.80	470.00	0	0	1	0	0	1	1	Positive ratio	0	1	2019-Ciuffi et al. [40]
29	5	5	0	44.80	3.27	300.84	5	3	0	2	0	1	1	0%	5	0	2021-Le Collen et al. [41]
30	4	3	1	46.50	—	—	0	4	0	0	0	2	1	0/1/2/3/4/5	3	1	2022-Popow et al. [42]
31–32	39	20	19	43.10	3.57	1240.57	0	0	39	0	0	2	0	0%	38	1	2020-Hu et al. [16] 2022-Hu et al. [43]

*Note: n*: the number of patients included in this study; M: male; F: female; HPT-JT: hyperparathyroidism-jaw tumor syndrome.

Abbreviations: APT, atypical parathyroid tumor; FIHP, familial isolated hyperparathyroidism; IHC, immunohistochemistry; PA, parathyroid adenoma; PC, parathyroid carcinoma; PH, parathyroid hyperplasia; PTH, parathyroid hormone.

**Table 2:** Clinical characteristics of the patients included in this study (*n* = 299 unless otherwise specified).

Characteristic	Total	Inconsistency group	Consistency group	*p* values
Male:Female (*n* = 290)	137:153	26:30	111:123	0.968
Average age at diagnosis (years) (*n* = 288)	47.91 ± 16.95	49.70 ± 16.25	47.49 ± 17.11	0.388
Serum calcium level (mmol/L) (*n* = 209)	3.28 ± 0.55	3.48 ± 0.58	3.24 ± 0.54	0.026^∗^
Serum PTH (pg/mL) (*n* = 195)	620.54 ± 703.21	853.02 ± 712.63	584.78 ± 697.00	0.070
Patients with familial history (Yes:No)	54:245	12:46	42:199	0.562
Pathological diagnosis (PC:non-PC)	163:136	41:17	122:119	0.006^∗^
*CDC73* mutation (Yes:No)	160:139	25:33	135:106	0.077
Parafibromin IHC (positive:negative)	131:168	25:33	106:135	0.903

*Note:* HPT-JT: hyperparathyroidism-jaw tumor syndrome.

Abbreviations: APT, atypical parathyroid tumor; FIHP, familial isolated hyperparathyroidism; IHC, immunohistochemistry; PA, parathyroid adenoma; PC, parathyroid carcinoma; PH, parathyroid hyperplasia; PTH, parathyroid hormone.

^∗^ *p* < 0.05.

**Table 3:** Possible reasons for inconsistent results of *CDC73* sequencing and parafibromin IHC staining in patients with parathyroid neoplasm.

Possible reasons	Inconsistent results	Consistent results	Univariate analysis *p* values	Multivariate analysis *p* values
Pathology (PC:non-PC)	41:17	122:119	0.006^∗^	< 0.001^∗^
Sequencing methods (NGS:non-NGS)	4:54	43:198	0.040^∗^	0.006^∗^
IHC antibody (sc33638:others)	36:22	131:110	0.288	0.888
Scoring criteria (total loss:partial loss)	14:43	40:201	0.160	0.946
Somatic mutation (positive:negative)	9:8	55:33	0.460	—

Abbreviations: IHC, immunohistochemistry; NGS, next-generation sequencing; PC, parathyroid carcinoma.

^∗^ *p* < 0.05.

## Data Availability

The datasets used and/or analyzed during the current study are available from the corresponding author on reasonable request.

## Supporting Information

The PRISMA_2020_checklist was provided in supporting file.
